# Development and Preliminary Validation of a Comprehensive Questionnaire to Assess Women’s Knowledge and Perception of the Current Weight Gain Guidelines during Pregnancy

**DOI:** 10.3390/ijerph13121187

**Published:** 2016-11-30

**Authors:** Holly Ockenden, Katie Gunnell, Audrey Giles, Kara Nerenberg, Gary Goldfield, Taru Manyanga, Kristi Adamo

**Affiliations:** 1 School of Human Kinetics, Faculty of Health Sciences, University of Ottawa, Ottawa, ON K1N 6N5, Canada; hockenden@cheo.on.ca (H.O.); kgunnell@cheo.on.ca (K.G.); agiles@uottawa.ca (A.G.); ggoldfield@cheo.on.ca (G.G.); TManyanga@cheo.on.ca (T.M.); 2 Heathy Active Living and Obesity Research Group (HALO), Children’s Hospital of Eastern Ontario (CHEO) Research Institute, Ottawa, ON K1H 8L1, Canada; 3 Department of Medicine, Cumming School of Medicine, University of Calgary, Calgary, AB T2N 4N1, Canada; kara.nerenberg@ucalgary.ca; 4 Department of Pediatrics, Faculty of Medicine, University of Ottawa, Ottawa, ON K1N 6N5, Canada; 5 Department of Psychology, University of Ottawa, Ottawa, ON K1N 6N5, Canada

**Keywords:** maternal health questionnaire, health questionnaire development, questionnaire validation, gestational weight gain knowledge, gestational weight gain perceptions, maternal health behaviours

## Abstract

The aim of this study was to develop and validate an electronic questionnaire, *the Electronic Maternal Health Survey* (*EMat Health Survey*), related to women’s knowledge and perceptions of the current gestational weight gain guidelines (GWG), as well as pregnancy-related health behaviours. Constructs addressed within the questionnaire include self-efficacy, locus of control, perceived barriers, and facilitators of physical activity and diet, outcome expectations, social environment and health practices. Content validity was examined using an expert panel (*n* = 7) and pilot testing items in a small sample (*n* = 5) of pregnant women and recent mothers (target population). Test re-test reliability was assessed among a sample (*n* = 71) of the target population. Reliability scores were calculated for all constructs (*r* and intra-class correlation coefficients (ICC)), those with a score of >0.5 were considered acceptable. The content validity of the questionnaire reflects the degree to which all relevant components of excessive GWG risk in women are included. Strong test-retest reliability was found in the current study, indicating that responses to the questionnaire were reliable in this population. The *EMat Health Survey* adds to the growing body of literature on maternal health and gestational weight gain by providing the first comprehensive questionnaire that can be self-administered and remotely accessed. The questionnaire can be completed in 15–25 min and collects useful data on various social determinants of health and GWG as well as associated health behaviours. This online tool may assist researchers by providing them with a platform to collect useful information in developing and tailoring interventions to better support women in achieving recommended weight gain targets in pregnancy.

## 1. Introduction

The rise in the prevalence of obesity is disproportionately affecting women of childbearing age [1]. A recent review of The Canadian Health Measures Surveys indicated that 48.2% of Canadian women are at a weight higher than considered healthy (i.e., body mass index (BMI) ≥ 24.9), which is up from 44.6% in 2001 [2]. As BMI has increased among the general population over time, so too has the number of overweight (OW) or obese (OB) women presenting for obstetric care, causing additional challenges and concerns for health care providers (HCP) [3]. Compared to those with a normal BMI, women who enter pregnancy already OW or OB are more likely to gain weight in excess of evidence-based guidelines [4], which can lead to poor antenatal and postpartum outcomes [5]. The weight gain targets for pregnancy, released by the Institute of Medicine (IOM), are based on pre-pregnancy BMI and were developed with the goal of reducing the number of pregnant women gaining excessive weight and experiencing adverse health consequences as a result [6].

Since these guidelines were updated in 2009, it has been reported that over 60% of pregnant women continue to exceed weight gain targets [7]. This is important as GWG within recommended guidelines has been associated with healthy pregnancy and positive clinical outcomes [5]. Inadequate GWG has been linked to an increased risk of preterm birth and delivering an infant with fetal growth restriction in some studies [7], whereas maternal pre-pregnancy obesity and excessive GWG are independently associated with increased birth weight, gestational diabetes (GDM), C-section and giving birth to an infant who is large for gestational age (LGA), and macrosomia [6,8].

The high prevalence of excessive GWG and its potential to contribute to adverse outcomes, underscores the importance of developing tools that will help HCP identify strategies to guide women in achieving optimal weight gain during pregnancy. Despite a large body of research describing the health complications related to excess GWG [8,9,10], only a small number of studies have assessed women’s knowledge about the updated IOM GWG guidelines [11,12]. To our knowledge, there are no published, validated tools developed to collect information on women’s perceptions of the current IOM guidelines or to examine reported barriers and facilitators contributing to GWG. Exploring women’s perceptions regarding these issues is important in order to assess the knowledge gaps regarding sub-optimal GWG, to discover the potential psychosocial risk factors and assist practitioners in helping women achieve the IOM recommendations [11,13,14,15,16].

In contrast to the minimal data available on women’s knowledge and perception of the effects that diet and exercise may have on pregnancy weight gain, there is documented evidence supporting the overall effects of energy intake and expenditure on weight gain and loss during pregnancy [17]. For example, Health Canada’s Healthy Weights Report states that healthy eating and physical activity (PA) practices are important for both weight management and general health [18], and other studies provide evidence to demonstrate that physical activity (PA) during pregnancy is beneficial for both mom and the baby [19,20]. Dietary guidelines have been developed that outline energy and nutrient intakes that have been shown to be most beneficial for each stage throughout pregnancy [21]. A recent Cochrane review on GWG interventions (diet, exercise, or both) reported success in reducing the risk of excessive GWG by 20% overall (average risk ratio (RR) 0.80, 95% confidence interval (CI) 0.73 to 0.87; participants = 7096; studies = 24; I^2^ statistic = 52%) [22].

Although the current IOM guidelines have been widely available, recent literature suggests that women are not being appropriately counseled on GWG targets [23,24]. Specifically, it was reported that women of healthy weight are more likely to be given information in accordance with the recommendations (86.8%) than women who were overweight (12.5%) or obese (26.3%) [25]. It is currently unknown whether women who are given appropriate guidance during pregnancy and pre-conception still exceed the recommended GWG. If this is the case, further research on women’s perceptions and self-efficacy relating to the IOM weight targets is needed because perception has been shown to affect motivation and, thus, success for weight management [26]. Subsequently, such information might be helpful in developing strategies on how best to provide information to women and also develop much needed clinical interventions that target barriers to weight management in pregnancy [24,25,27].

The current study had two objectives: (1) develop a self-administered, web-based, comprehensive questionnaire (i.e., online survey) to examine factors influencing weight gain among pregnant women that can be incorporated into both clinical practice and future research, and (2) establish initial content validity and evaluate test-retest reliability of the questionnaire among a sample of pregnant women and mothers ≤5 years postpartum. We hypothesized that responses to the questionnaire will demonstrate validity as measured by content validity via an expert panel review and pilot testing items with the target population, as well as reliability assessed by a 14 day test-retest sub-study period.

## 2. Materials and Methods

### 2.1. General Methods

All subjects gave their informed consent for inclusion before they participated in the study. The study was conducted in accordance with the Declaration of Helsinki, and the protocol was approved by the Ethics Committee of the Children’s Hospital of Eastern Ontario Research Institute, Research Ethics Board (CHEOREB#09/03E) and the University of Ottawa Ethics Committee (14/183X).

The survey was built using branching logic to avoid redundancy and to capture participant-specific information. For example, women who reported no current pregnancy were directed via branching logic past the items on current pregnancy to the past pregnancy section only. As part of the validation study, for items where “other” was a chosen answer option, an ensuing question was available for a textual description. The only question where participants entered typed information for “other” was in response to an item addressing dietary information from their HCP. As a result, an item was added to include the options listed from participants (i.e., the options “increase the amount of fibre” or “decrease the amount of fibre” were added). The content validity team (expert panel) also changed the order of some indicators to allow for better flow of the questionnaire (i.e., vitamin, smoking and alcohol intake items were moved from health practices to the diet construct), which has been relabeled to “diet and smoking construct” for validated survey studies.

#### 2.1.1. Target Population

Women who were currently pregnant or reported a live birth in the year after the most recent IOM guidelines were released (2009) were recruited between February and May 2015. Social media websites (i.e., Facebook page and twitter postings) were used to promote the study to the target population. The postings included a link to the REDCap^TM^ website that housed the questionnaire. Women who clicked the link were directed to the consent form for more information on the study and, after completion of the consent form, were able to begin the questionnaire. In addition to pregnant women, postpartum women who had given birth since the release of these guidelines (within the last five years at the time of completion) were included. 

Pregnancy represents a very defined time in a woman’s life course and there are data to suggest that even 32 years after delivery, maternal recall is highly correlated with documented pre-pregnancy height and weight (*r* = 0.95), obstetric complications (*r* = 0.74), birthweight (*r* = 0.94), breastfeeding (*r* = 0.89) and various other health behaviours (*r* = 0.73–0.87) [28]. Including postpartum women allowed us to gather information on awareness of GWG guidelines over the course of pregnancy, whether or not one’s HCP had discussed weight gain recommendations or other pregnancy-related recommendations and if a woman’s behaviour might have changed had she been informed or counseled accordingly.

#### 2.1.2. Sample Size

A priori power analysis was calculated using G*Power. Based on alpha = 0.05, power = 0.80, and specifying a moderate correlation = 0.53, the required sample size for the current investigation was *n* = 67. Study data were collected and managed using REDCap^TM^, a secure, web-based application designed to support data capture for research studies.

### 2.2. Questionnaire Development

The development of the comprehensive questionnaire was conducted in eight steps, as detailed below.

#### 2.2.1. Literature Review and Identification of Variables

A literature review was conducted to identify existing validated questionnaires that evaluated any of the following: barriers and facilitators to weight management in pregnancy and women’s perceptions of the updated IOM guidelines. Of note, we did not find any questionnaires that used the updated GWG guidelines [5].

#### 2.2.2. Literature Review to Identify a Psychological Theoretical Framework

We selected Social Cognitive Theory (SCT) to develop our comprehensive maternal health questionnaire related to GWG and associated health behaviours [29]. SCT describes predictive and modifiable factors associated with behaviour change [30]. The core concept in SCT is reciprocal determinism, which refers to the dynamic and shared interaction of person (learned experiences), environment (social context), and behaviour (responses to information and advice), which, in combination, produce a response [29]. Although there are multiple constructs within SCT, the following five variables were highlighted in health behaviour change studies and considered modifiable: (1) outcome expectations (can be influenced by the outcome or behaviours of others); (2) perceived barriers to achieving wanted behavior; (3) self-efficacy (one’s perception about how effective behaviour can control events affecting them); (4) locus of control (belief whether external (the environment) or internal (one’s own personality, choices, thoughts) factor are controlling one’s behaviour); and, finally, (5) social environment (social pressures experienced) [29,31]. 

#### 2.2.3. Development of a Draft Questionnaire

A draft questionnaire containing a pool of 110 items was developed based on key psychosocial predictors of GWG that were identified in the literature review and guided by components of the SCT (self-efficacy, locus of control, perceived barriers, outcome expectations, and social environment). Pregnancy-specific weight gain interventions have shown that knowledge of lifestyle recommendations (including GWG, diet, and PA) and health practices were also key predictors in amount of weight gained while pregnant [32] and, thus, were also included as a constructs. Next we examined the 110 original items (approximately 50 on current pregnancy and 60 on previous pregnancy), divided within the seven constructs, and removed duplicate items and any item that was ambiguous. Additional questions addressing post-pregnancy health plans (i.e., breastfeeding practices) were added, resulting in approximately 80 items total (30 on current pregnancy and 50 on past pregnancy). The questionnaire was structured so that participants who were currently pregnant answered questions based on current behaviours and perceptions, while postpartum women answered the questionnaire based on retrospective behaviours and perceptions during their most recent past pregnancy. Women who reported both currently being pregnant and given birth after the year 2009 (when the updated guidelines were published by the IOM) were prompted to complete both pregnancy and postpartum questions (all questions related to current pregnancy were asked first, followed by those relating to past pregnancy).

Most questions on perceived weight and GWG factors were unique to this study. Some items were developed based on existing questionnaires that were now considered out of date (i.e., used the 1990 IOM guidelines). For example, the *Pregnancy and Weight Gain Attitude Scale* (PWGAS) [33], comprises 18 items about weight-related attitudes (15 items) and behaviors (three items) during pregnancy and refers respondents to the outdated IOM guidelines and terminology. As such, these questions were adapted to reflect the 2009 GWG targets for the current study [33]. The *Pregnancy Experience Scale* (PES) [34], which was developed to measure maternal appraisal of exposures to 41 potential hassles specific to pregnancy, only included one item that addressed weight. Therefore, this item was adapted for the present questionnaire. PA behaviour was assessed with questions based on the Leisure Time Exercise Questionnaire [35]. The items were adapted to assess the number of times per week the participant engaged in light, moderate, and vigorous exercise rather than number of activity bouts. The final version of the questionnaire had two sections and six constructs with 60 items in total (see Table 1). It incorporated all listed predictors of weight gain in pregnancy as well as specific behaviours. (Section 1: Inclusion/Exclusion; Section 2: Demographics; Construct 1: Health Practices; Construct 2: Pregnancy Weight; Construct 3: Physical Activity; Construct 4: Diet; Construct 5: Pregnancy Intentions and/or Pregnancy Practices; and Construct 6: Diet and Weight Gain Perceptions).

#### 2.2.4. Expert Panel Feedback

An expert panel (*n* = 7) was established and comprised of a clinical psychologist with specialization in health behaviour, a qualitative researcher in women’s health with expertise in socio-cultural aspects of behaviour, a physiologist with expertise in nutrition and PA in pregnancy, a clinician specializing in internal medicine/obstetrics, two maternal health research experts, and a pregnant woman who was also a recent mother. During the development phase, the expert panel provided guidance on questionnaire items to improve content validity. Once content was approved by the expert panel, the questionnaire was created and study data were collected and managed using REDCap^TM^ electronic data capture tools hosted at the CHEO RI (Ottawa, ON, Canada) [36]. 

#### 2.2.5. Pilot Study and Questionnaire Revision

A preliminary version was pilot-tested on a convenience sample of five consenting women (two currently pregnant and three who had been pregnant after May 2009, all single fetus pregnancies). For the pilot study the questionnaire took approximately 15–25 min to complete, depending on whether the participant was currently pregnant, postpartum, or both. The questionnaire was available in English only. Women provided feedback on any misleading and confusing questions by email to the author. Participants were particularly adamant about noticeable redundancies in the questionnaire being a barrier to completion. Suggestions from the pilot participants included adding options for types of diet, simplifying wording to some questions and removing potentially repetitive questions. All reported feedback was emailed to the lead author, documented, and addressed. Questions were removed or adapted based on these suggestions.

#### 2.2.6. Questionnaire Validity

Content validity was established through incorporation of variables/questions from the literature review in combination with the suggestions from the content experts who contributed to the development of the questionnaire (via an expert panel). By addressing content validity we also ensured that less relevant variables were excluded to reduce participant burden [37].

#### 2.2.7. Test Re-Test Reliability (Using a Two-Time Point Study)

Women who identified as either (1) currently pregnant or (2) birth after May 2009 with a single fetus were eligible to participate. Women currently pregnant were asked to complete approximately 30–40 items, while women who reported giving birth after May 2009 completed 45–55 items. Participants were prompted to accept informed consent via an online form before completing the self-administered computer-based questionnaire.

Based on review of previously validated questionnaires in the area, Test-retest periods ranged from one week to one month. We chose to perform the test-retest reliability of our questionnaire using a two-week (14-day) interval. Our expert panel believed this would ensure that all of our participants would click their retest link and finalize their responses within a period of one-month from their initial response. Participants were contacted (via auto response email, with a link to the retest survey) and prompted to complete the same questionnaire on the same day of the week. Two reminder e-mails were sent to subjects over six days to optimize response rates. Data were exported from REDCap^TM^ into SPSS (SPSS Inc., Chicago, IL, USA) for analysis.

#### 2.2.8. Final Version of Questionnaire

Based on the results of the previous steps identified above, we created the final questionnaire that was used for the study.

## 3. Data Collection and Management

**Figure 1:**
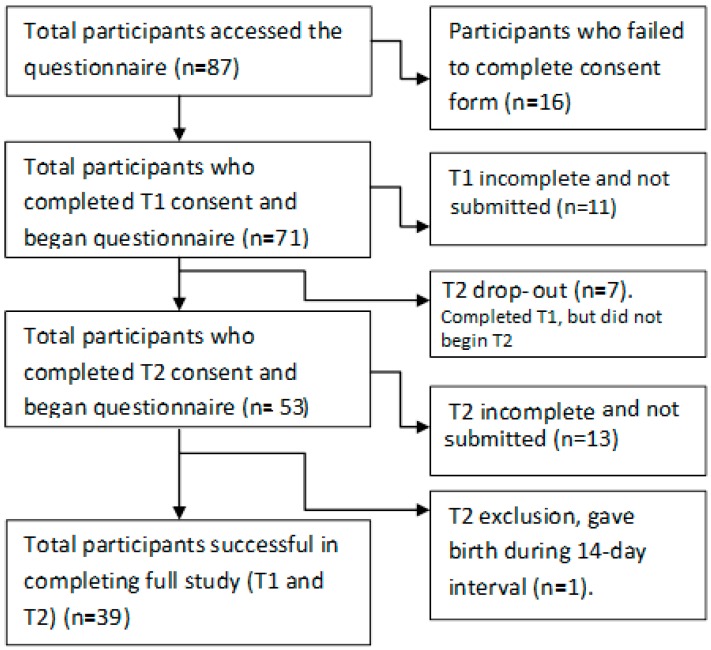
Outline of participants in each phase of test-retest validation study (participation rate); T1 = test 1, T2 = re-test 2.

A total of 87 women clicked on the public survey link and viewed the online questionnaire consent form. Although no one refused consent, 16 did not complete the consent process. The total number of participants who completed test 1 (T1) was 60 (*n* = 11 incomplete T1 and *n* = 7 drop out after T1 complete). The total number of women who completed both T1 and Test 2 (T2) was *n* = 39 (*n* = 14 incomplete at T2). One study participant gave birth within the 14 day test-retest interval and was, therefore, excluded from the study. The number of participants included in the final validation analysis was *n* = 39 (Figure 1).

## 4. Statistical Analysis

All statistical analyses were carried out using SPSS/PC + (SPSS Inc., Chicago, IL, USA). Single-measure intra-class correlation coefficients (ICC) were used to evaluate the test–retest reliability within the 14-day interval. ICC values were interpreted as very high (ICC > 0.9), high (ICC > 0.75), or moderate (ICC between 0.5 and 0.75) [30]. The ICC is used for measures with several categories or for linear measures, and is appropriate for use in samples that include non-independent data [37,38]. Another commonly used test to assess reliability of various health questionnaires is the Pearson’s correlation (*r* value) of the data between test and retest [39]. The *r* value was also calculated for all questions to use as a comparison to the ICC. According to Field et al., large correlation coefficients, defined as 0.5 or greater, indicate that the reliability is high [38]. Statistical significance for this study is based on the *p* < 0.05 level for all analyses and specifying a one tailed test of significance. Questions with multiple response options were not included in the ICC or Pearson correlation analysis given that these analyses require the selection of only one response option.

## 5. Results

Participants included 71 women (32% currently pregnant and 68% recently postpartum (since 2009)). The most common reported demographics are reported in Table 2.

### 5.1. Test-Retest Reliability

Pearson’s *r* and ICC values are presented for all items (Table 2). Two correlation measures were used to show both similarity in the sum of responses to an item between the two time points (test and retest), and the proportion of individual respondents whose responses were the same on both occasions [22]. Correlation coefficients were considered acceptable for most items (>0.5) in the questionnaire (Table 3). Table 3 shows the correlation coefficients, Pearson’s *r* and ICC, for each category of variables measured.

Test-retest bivariate correlations for all items within the Self-Efficacy construct (two items) and many within the Social Environment construct (10 items) were >0.5 for Pearson *r* and ICC. Despite the low test-retest reliability of some items, we decided to retain the questions for the final version of the questionnaire because the statistical power of this question was low due to small completion sample (*n* = 16), which could have led to an attenuated test-retest value. It is possible that with a larger sample size and more statistical power, the test-retest correlation for this item could have reached recommended levels (>0.05 for Pearson *r* and >0.5 for ICC). Additionally, the self-efficacy questions were important in fulfilling the objective to collect information on women’s confidence in the ability to exert control over their own behaviour and social environment.

There were six items analyzed for the Locus of Control construct. All but one question within the construct were deemed acceptable (*r* and ICC > 0.5). The question “Why would you feel most comfortable gaining this amount while pregnant?” (*r* = 0.215, *p* = 0.201, ICC = 0.545, *p* = 0.201) reported a low *r* value but significant ICC. This question was kept as part of the questionnaire as the statistical power of this question may be low due to the small sample (*n* = 10).

The Social Environment construct included a total of ten items. Six items demonstrated a range of reliability (*r* values ranged from 0.221–0.706 and ICC values ranged from 0.220–0.702). The remaining four items produced low reliability values; these items were either removed or reworded.

All items within the Outcome Expectations construct (two items) showed significant test-retest reliability (*r* = 0.618, 0.712, and ICC = 0.664, 0.711 with a *p* ≤ 0.001 for both). All items were retained within the final questionnaire. The Health Knowledge construct included 13 items. Ten items demonstrated high reliability between survey administrations (between a perfect *r* = 1, ICC = 1 and *r* = 0.615, ICC = 0.607, *p* = 0.04). Although four items had low test-retest reliability, the overall mean of the construct (*r* = 0.625, ICC = 0.621) was good and, therefore, a decision was made to retain the items for future validation studies.

Test-retest bivariate correlations for the Health Practice/Plans construct (21 items) ranged from poor to a perfect correlation (ICC 0.361 to 1.0) and low to perfect (*r* values range from 0.417–1.0). There were two items that were found to have poor reliability, (1) “How much more/less are you eating (compared to usual pre-pregnancy amount) during your pregnancy?” (*r* = 0.417, ICC: 0.361 *p* = 0.202); and (2) “Comparing your current weight to your weight one year pre-pregnancy, how has your weight changed?” (*r* = 0.256, ICC = 0.254, *p* = 0.378). As a whole, the health knowledge construct was considered significant in test-retest consistency. It was suggested by the questionnaire development team (expert panel) to keep these two items with low reliability as answers may have changed to these items as a result of the 14-day retest window (i.e., weight related answers may have changed because, presumably, women would be gaining more weight over the 14 days while pregnant). These items will be further analyzed in future validation studies. Due to a low sample size (*n* = 2) for the perceived barriers/facilitators (two items) construct, a test-retest score was not calculated.

## 6. Discussion

The aim of our study was to develop a comprehensive questionnaire that can be used to examine influential factors of weight gain among pregnant women to allow for use in future research and clinical settings to identify contributors to GWG (i.e., positive or negative contributing factors to weight management). Our second objective was to evaluate test-retest reliability of response to the questionnaire among a sample of pregnant women and recent mothers. The calculated reliability scores (*r* vales and ICC) of most constructs were considered moderate to high. The lowest reported scores were within the social environment construct, and the highest scores were from the health practice/plans construct. We have provided initial evidence for test-retest reliability and content validity for this comprehensive questionnaire. More research, however, is needed to examine other aspects of reliability and validity with larger sample sizes to enhance the psychometric properties of the developed instrument.

A strength of this study is the use of rigorous methodology in the development of the comprehensive questionnaire [40,41] including the incorporation of an expert panel to assess content validity. A benefit of this comprehensive questionnaire, especially within the field of maternal health research (for pregnant women or recent mothers), is that a self-administered, remotely-accessed questionnaire can be completed at home, at any preferred time, thereby allowing for easy, fast recruitment and data collection and potentially greater participation. The questionnaire can be used in any setting from rural to urban communities [42], both of which have been shown to be at increased risk of exceeding the GWG guidelines for reasons yet to be explained [43]. This questionnaire could be applied in several settings, ranging from future health research, clinical use, or use in population screening for excessive GWG risk. Nevertheless, given that validation is considered an ongoing process; more research is needed to determine the most efficient way to use this tool and to further examine the validity of response to the questionnaire.

There are several limitations to be considered in our preliminary study. One limitation is the low response rate and the large attrition rate (i.e., those who originally completed initial consent (T1) vs. those who finished the final retest (T2)). This may be related to participants not fully understanding the need to complete the second questionnaire and, thus, ignoring the prompts and messages. Participants did not respond to all questions resulting in the numbers identified in Table 3. Given the final sample size we combined relevant responses from pregnant and postpartum women to have sufficient power. A second limitation is that the questionnaire could be assessed both during and after pregnancy (in two different versions) and, therefore, the timeline of use must be clearly defined in future studies. A third limitation is that the questionnaire is only available in the English language and we do not know how well this questionnaire will perform in English-as-a-second-language speakers or in people with more limited health literacy (i.e., the ability to access, comprehend, evaluate and communicate information as a way to improve health [44]). To reach a wider population, questionnaire items should undergo rigorous language adaptation procedures and responses to the questionnaire in alternative languages should be validated. Similarly, responses to the questionnaire from people with different degrees of health literacy or socioeconomic status should be validated. Although the recruitment procedures used were open to those of any ethnicity, education, or SES, the resulting sample population included mostly white, educated, Canadian women, which may limit the generalizability to a broader population and interpretation of the results. It must also be noted that “postpartum” women completed the questionnaire up to five years following pregnancy; thus, their responses may have been subject to recall bias. However, as mentioned previously, maternal recall of pregnancy-related characteristics and behaviours is quite remarkable many years postpartum [28]. In fact, there are data to suggest women recall GWG quite reliably within one year of delivery (*r* = 0.99) [45], between 4 and 12 years post-pregnancy (*r* = 0.63) [46] and, even 30+ years after delivery, maternal recall of GWG is modestly correlated (*r* = 0.42) with documented GWG [28]. Finally, the small sample size for some constructs and items (i.e., perceived barriers and facilitators construct) is a limitation. Partial participation in these questions may have led to inconclusive test-retest results for those variables. As such, future studies should use larger, more representative samples and longitudinal data to establish stronger reliability.

We acknowledge that the *EMat Health Survey* is lengthy. It was designed to be comprehensive in diverse aspects of maternal health related to gestational weight gain. Initially, in order to reduce the length of the questionnaire, only barriers to managing weight, diet, and adequate PA were included. After consulting with the expert panel it was agreed that in order to ensure a more accurate depiction of the psychology behind women’s health behaviours, facilitators should also be considered. Facilitator questions were added to address this concern (i.e., you answered “most of the time” or “always” to feeling confident about being able to stay within the weight guidelines suggested by your health care provider. Why do you feel this way?). Additional self-efficacy and locus of control questions were added to include more in-depth questions around these constructs regarding weight gain in pregnancy. These added questions were considered valuable by the expert panel and the project team. As suggested by experts, questions were added to address social norms, as this is an important component of the SCT. Social impact has been studied and shown to greatly affect weight beliefs and practices in adults. The impact they have on weight gain in pregnancy, specifically, is an area that has limited data. In addition, during the development of the questionnaire, a decision was made a priori and supported by the expert panel to remove any redundant questions, to reduce the length and repetitiveness of the survey, at the expense of being able to create the subscales. Future studies should evaluate how the length of the questionnaire affects women’s participation and whether shorter versions can be developed, tested for validity and reliability, and used instead of this comprehensive version.

The length of the survey may have been the cause of a high loss to follow-up rate between test and retest (45% loss to follow-up). One approach to increasing response and reducing loss to follow-up is the use of incentives, which has been effective at increasing response rates in other surveys [47]. Incentives (such as gift vouchers or lottery participation) have been found to almost double the odds of response to electronic surveys [47] and may be useful in future studies.

Despite these limitations, a strength of this study is the novel basis of our questionnaire, as it is the first known questionnaire aimed to examine women’s perceptions of the IOM GWG guidelines, the relevance of pre-pregnancy weight and GWG, whether or not they were provided with pertinent information regarding evidence-based pregnancy recommendations and to identify both the barriers and facilitators women report regarding weight management in pregnancy. The questionnaire is self-administered and can also be accessed on-line, which are additional strengths and add to the diverse future potential applications of the questionnaire in health research and clinical practice. Maternal care providers should be discussing the GWG guidelines with all women, regardless of pre-pregnancy BMI [48]. Raising awareness regarding the benefits of meeting guidelines and having sensitive conversations to assist women in decision making regarding pregnancy-related behaviours may lead to more appropriate weight gain, and a reduction in delivery complications. This survey information may provide health care advocates and policy makers with important information to guide the development and implementation of clinical interventions to target GWG.

## 7. Conclusions

In conclusion, this online questionnaire to assess women’s perceptions of the current IOM weight gestational weight gain guidelines demonstrates preliminary content validity and test-retest reliability. It is a comprehensive questionnaire that can be implemented in diverse health care environments to increase the detection of women at risk of excessive GWG and address the barriers women report in pregnancy. This study fills gaps in the existing literature related to the lack of reliable and valid instruments available to gather information about the perceptions of women regarding weight gain in pregnancy, as well as knowledge of, and engagement in, healthy prenatal lifestyle behaviours. While comprehensive questionnaire strategies, this given the high rates of maternal obesity and excessive GWG and their consequences for obstetrical care and overall population health, for clinical integration further studies of the determinants of GWG are urgently needed. Future research would also warrant collecting information on the perceptions of both the study population (pregnant and postpartum women), as well as health care professionals on the usefulness and practicality of this tool within clinical practice. In addition, looking at women who are currently pregnant independent of, as well as in comparison to, postpartum women, would be a second future research direction that would be beneficial.

This questionnaire can be used to help better understand what is causing women to gain more weight than recommended in pregnancy and serve as an early step in addressing obesity in young women of reproductive age.

## Figures and Tables

**Table 1:** Questionnaire section and construct descriptions.

Section 1: Inclusion/Exclusion	Over 18 years old Currently pregnant Birth of child after May 2009 (i.e., in the last 5 years) Single fetus pregnancy
Section 2: Demographics	Age Height (ft./in or cm) Weight (kg or lbs.) (if currently pregnant, women were asked for both current weight and pre-pregnancy weight) Gestational Age (if pregnant) Location of received healthcare (e.g., country/province/state of healthcare) Household income Ethnicity Marital status Education
Construct 1: Health Practices (current and/or past pregnancy) (nine items)	Health care location (hospital, home, clinic) (two items) GWG counseling by HCP (two items) Alcohol and tobacco use (two items) Birth center preference (one item) Medical conditions or medications (two items)
Construct 2: Pregnancy Weight (current and/or past pregnancy) (eight items) Adapted from Palmer, 1985 [33]	Weight self-perception (two items) Gestational weight gain recommendation knowledge (two items) Social network advice (two items) Facilitators and barriers to pregnancy weight management (two items)
Construct 3: Physical Activity (PA) (current and/or past pregnancy) (12 items) Type of PA (three items) were adapted from Godin, 1985 [35]	Type of PA (three items) Current PA plans and practices (four items) Facilitators and barriers to PA in pregnancy (two items) Health care provider advice (one item) Social network advice (two items)
Construct 4: Diet (current and/or past pregnancy) (11 items)	General diet habits (four items) Pregnancy diet recommendation knowledge (three items) Any specific diet (two items) Health care provider advice (one item) Social support advice (one item)
Construct 5: Pregnancy Intentions (for current pregnancy) and/or Pregnancy Practices (for past pregnancy) (eight items)	Birth plans (two items) Breastfeeding or infant feeding plans (two items) Health care provider breastfeeding support (two items) Prenatal education class participation (two items)
Construct 6: Diet and Weight Gain Perceptions (current and past pregnancy) (14 items) one item adapted from DiPietro (2002) [33]	Locus of control (of weight gain in pregnancy) (two items) Locus of control of diet (two item) General weight gain in pregnancy questions (two items) Perceived success of attaining IOM GWG recommendations (three items) Feelings regarding facilitators and barriers to weight discussions with health care provider (two items) Postpartum weight retention (for those who reported past pregnancy only) (three items)

**Table 2:** The most common reported demographics.

Question	Most Common Answer	% Participants
Current age	30–34 years old	44.9%
Gestational age (for those who were pregnant)	25–29 weeks	26.7%
Nationality	Canadian	92.0%
Ethnicity	White	97.4%
Marital status	Married	89.6%
Education	Post-secondary graduate	79.1%
Employment	Employed full time	79.5%
Household Income	$150,000+/year	34.6%

**Table 3:** Test-retest bivariate correlations for all constructs.

Construct Name (Number of Items Analyzed in Construct) and Listed Items	*N* Test	Mean (SD) Test	*N* Retest	$\bar{x}$ (SD) Retest	Pearson Correlation (*r* Value)	*p* Value (Sig 1-Tailed)	Test Re-Test Reliability ICC (95% CI)
Self-efficacy (2)							
Did you feel confident about being able to stay within the weight gain limits provided for your past pregnancy?	18	4.44 (0.784)	16	4.50 (0.516)	0.472	0.075	0.472 (−0.033, 0.785)
Did you make a focused effort to stay within the weight gain limits given to you by your health care provider?	18	3.11 (1.530)	16	2.75 (1.483)	0.397	0.142	0.396 (−0.126, 0.746)
Locus of control (6)							
Do you worry that you may gain too much weight while pregnant?	24	1.83 (1.167)	24	1.88 (1.191)	0.950 **	<0.001	0.950 *** (0.878, 0.980)
I would feel MOST comfortable gaining the following amount during my pregnancy:	4	4.00 (1.155)	8	3.88 (0.835)	0.707 *	0.293	0.667 ** (−0.511, 0.974)
I would feel MOST comfortable gaining the following amount if I were to become pregnant:	21	4.14 (1.352)	26	3.85 (1.287)	0.749 *	<0.001	0.545 * (0.147, 0.791)
Why would you feel most comfortable gaining this amount while pregnant?	28	2.11 (1.571)	26	1.54 (1.208)	0.215	0.201	0.208 (−0.120, 0.496)
I am able to control the amount of weight that I gain while pregnant	38	2.68 (1.016)	37	2.62 (0.982)	0.549 *	0.012	0.646 * (0.326, 0.833)
If I am healthy and exercise, I can control my weight while pregnant.	38	2.26 (1.131)	37	2.41 (1.040)	0.521 *	0.001	0.520 * (0.240, 0.720)
Outcome Expectations (2)							
Do you think that women should be careful about gaining too much weight during pregnancy?	38	3.13 (1.119)	37	2.70 (1.077)	0.618 *	<0.001	0.617 * (0.370, 0.783)
As long as you are eating a well-balanced, healthy diet, do you feel it shouldn‘t matter how much weight you gain while you‘re pregnant?	38	4.08 (1.075)	37	3.78 (1.134)	0.712 *	<0.001	0.711 * (0.506, 0.840)
Social Environment (10)							
Have any of your friends or family members been pregnant in the past 5 years?	38	2.50(.604)	37	2.43 (0.835)	0.231	0.168	0.220 (−0.108, 0.505)
If yes, how much weight, on average, did your family members gain while pregnant?	23	4.91 (2.811)	28	5.61(3.059)	0.334	0.129	0.332 (−0.094, 0.656)
If yes, how much weight, on average, did your friends gain while pregnant?	36	5.08 (2.792)	29	5.79 (2.846)	0.651 *	<0.001	0.651 * (0.373, 0.822)
How do you feel about the amount of weight they gained while pregnant?	36	2.86 (0.351)	37	2.89 (0.315)	0.623 *	<0.001	0.620 * (0.366, 0.789)
Did you receive advice from your family or friends about how much weight to gain while pregnant?	38	3.55 (0.921)	37	3.38 (1.089)	0.341	0.039	0.337 (0.018, 0.593)
Whose advice do you trust the MOST (in regards to weight gain while pregnant)?	38	3.95 (0.324)	36	3.78 (0.760)	0.401	0.015	0.295 (−0.033, 0.565)
Do you feel it is acceptable to gain as much weight as you want while you are pregnant?	38	2.50 (1.157)	37	2.30 (1.127)	0.555 *	<0.001	0.555 * (0.285, 0.743)
How comfortable do you feel discussing your weight and/or weight gain during pregnancy with friends or family members?	38	2.97 (1.000)	37	3.11 (0.774)	0.221	0.188	0.221 (−0.106, 0.506)
How comfortable do you feel discussing your weight and/or weight gain during pregnancy with your health care provider?	37	3.32 (0.933)	37	3.32 (0.944)	0.706 *	<0.001	0.702 * (0.492, 0.834)
Whose advice do you trust the MOST (in regards to diet while pregnant)?	38	1.63 (1.478)	37	1.38 (1.139)	0.113	0.506	0.109 (−0.219, 0.415)
Health Knowledge/Knowledge of Recommendations (13)							
Thinking of your pre-pregnancy weight, what weight did you consider yourself?	12	2.17 (0.389)	11	2.18 (0.405)	1.000 ***		1.000 ***
Are you following Canada‘s Food Guide while pregnant?	12	3.25 (1.485)	11	3.82 (1.834)	0.615 *	0.044	0.607 * (0.048, 0.876)
Before this pregnancy, what weight did you consider yourself?	32	2.25 (0.508)	31	2.29 (0.588)	0.955 **	<0.001	0.947 *** (0.891, 0.974)
Did you follow Canada‘s Food Guide while pregnant?	32	3.66 (0.787)	31	3.81 (0.833)	0.753 *	<0.001	0.753 ** (0.543, 0.874)
Do you currently follow a specific diet?	38	7.11 (2.024)	37	7.38 (1.754)	0.804 **	<0.001	0.795 ** (0.636, 0.889)
When you found out you were pregnant did you change your eating habits (either for the better or worse)?	31	2.68 (1.661)	31	2.58 (1.523)	0.482	0.007	0.481 (0.151, 0.714)
Did your eating habits change for better or worse?	12	3.33 (1.557)	11	3.55 (1.508)	0.489	0.006	0.486 (0.158, 0.717)
How much more/less did you eat during your first trimester of pregnancy?	32	3.56 (1.014)	31	3.39 (1.174)	0.692 *	<0.001	0.690 * (0.444, 0.840)
How much did you eat during your second trimester of pregnancy?	32	3.03 (1.121)	31	3.00 (1.211)	0.565 *	0.001	0.565 * (0.263, 0.766)
How much did you eat during your third trimester of pregnancy?	32	3.50 (0.984)	31	3.61 (0.715)	0.643 *	<0.001	0.609 * (0.324, 0.793)
Where did you find out about any pregnancy related calorie (kcal) change information?	32	4.41 (1.932)	31	4.23 (2.045)	0.170	0.369	0.170 (−0.197, 0.495)
How often have you been weighed by your health care provider during this pregnancy?	11	1.00 (0)	10	1.00 (0)	0.891 **	<0.001	0.887 ** (0.768, 0.947)
How often did your health care provider talk to you about weight gain and weight gain limits? (Please account for advice given during your past pregnancy only).	18	3.06 (1.514)	16	2.94 (1.569)	0.436	0.105	0.435 (−0.079, 0.767)
Health practices/plans (21)							
Why are you currently not taking a prenatal vitamin?	2	3.50 (3.536)	2	3.50 (3.536)	1.000 **		1.000 ***
If the choice was available, in what type of center would you want to give birth?	12	2.50 (0.674)	11	2.64 (0.674)	0.824 **	0.002	0.824 ** (0.471, 0.949)
How much more/less are you eating (compared to usual pre-pregnancy amount) during your pregnancy?	12	4.000 (0.739)	11	3.91 (0.701)	0.417	0.202	0.361 (−0.271, 0.776)
Have you consumed any alcoholic beverages at any point during this pregnancy?	12	6.58 (0.900)	11	6.36 (1.206)	0.871 **	<0.001	0.844 **
Have you smoked cigarettes at any point during this pregnancy?	12	7.00 (0)	11	7.00 (0)	-	<0.001	-
Comparing your current weight to your weight 1 year pre-pregnancy, how has your weight changed? Do you weigh:	20	2.15 (1.814)	18	2.89 (2.111)	0.256	0.378	0.254 (−0.299, 0.679)
How often do you participate in high intensity workouts per week (heart beating rapidly; e.g., running, jogging, hard swimming or hard cycling)? (During this pregnancy)	12	2.33 (1.875)	10	2.60 (1.578)	0.615 *	<0.001	0.915 *** (0.717, 0.976)
Additional past pregnancy health practice questions							
In what type of centre did you give birth?	38	1.61 (0.679)	37	1.57 (0.647)	0.721 *	<0.001	0.720 * (0.519, 0.845)
What was the method of delivery? (C-section, vaginal, VBAC)	32	2.84 (0.515)	31	2.84 (0.523)	1.000 **	<0.001	1.000 ***
Did you smoke cigarettes at any point during pregnancy?	32	6.63 (0.660)	31	6.42 (1.232)	0.852 **	<0.001	0.712 * (0.478, 0.852)
How long after birth did you begin breastfeeding?	32	6.81 (1.061)	31	6.84 (0.898)	1.000 **	<0.001	0.984 *** (0.966, 0.992)
What was your child fed after birth?	32	1.84 (1.221)	31	1.84 (1.214)	0.957 **	<0.001	0.956 *** (0.910, 0.979)
How many months did you exclusively breastfeed (number of months)?	32	1.22 (0.553)	31	1.32 (0.702)	0.771 *	<0.001	0.766 ** (0.565, 0.881)
How many months did you exclusively formula feed (number of months before introduction of other foods)?	32	4.94 (3.212)	30	5.40 (3.081)	0.935 **	<0.001	0.934 *** (0.864, 0.968)
How often did you participate in medium intensity workout(s) per week (not exhausting; e.g., fast walking, easy cycling or dancing)?	32	0.78 (1.184)	31	0.61 (1.022)	0.646 *	<0.001	0.612 * (0.328, 0.794)
How often did you participate in high intensity workout(s) per week (heart beating rapidly; e.g., running, jogging, hard swimming or hard cycling)?	32	3.00 (1.867)	31	2.97(1.741)	0.832 **	<0.001	0.829 ** (0.671, 0.915)
How many times per week did you exercise during your pregnancy? This includes any type of physical activity.	32	1.41 (0.798)	31	1.45 (0.810)	0.975 **	<0.001	0.974 *** (0.945, 0.987)
How often did you participate in low intensity workout(s) per week (minimal effort; e.g., easy walking, yoga or golf)?	31	1.58 (1.544)	31	1.71 (1.596)	0.766 *	<0.001	0.765 ** (0.559, 0.882)
During a typical 7-Day period (one week), how often do you engage in regular activity that is long enough to work up a sweat (heart beats rapidly)?	37	2.97 (1.213)	37	3.08 (1.256)	0.869 **	0.002	0.868 ** (0.523, 0.969)
If yes, how long did it take to return to your pre-pregnancy weight?	25	1.36 (0.490)	23	1.43 (0.590)	0.535 *	0.001	0.532 * (0.256, 0.728)
If no, Are you satisfied with your current postpartum weight?	10	2.40 (0.843)	12	2.25 (0.754)	0.708 *	<0.001	0.696 * (0.396, 0.861)

*: *p*-Value < 0.05; **: *p*-value < 0.01; ***: *p*-value < 0.001.

## Abbreviations

The following abbreviations are used in this manuscript:

GWG	Gestational Weight Gain
BMI	Body Mass Index
IOM	Institute of Medicine
SES	Socioeconomic Status
OW	Overweight
OB	Obese
HCP	Health Care Provider
ICC	Inter Correlation Coefficient
PA	Physical Activity
HBM	Health Belief model
PES	Pregnancy Experience Scale
PWGAS	Pregnancy Weight Gain and Attitude Scale
